# Selections and Abstracts

**Published:** 1916-10

**Authors:** 


### SELECTIONS AND ABSTRACTS

### ABSTRACT OF ARTICLE PUT IN NEW YORK MEDICAL JOURNAL SEPTEMBER 9, 1916, ENTITLED "SOME THOUGHTS ON PROSTATECTOMY."

By Henry H. Morton, M. D.

Clinical Professor of Genito-Urinary Diseases in the Long Island College Hospital; Genito-Urinary Surgeon to Long Island College and Kings County Hospitals, and the Polhemus Memorial Clinic, Etc., Brooklyn, N. Y.

Cases of hypertrophied prostate may be divided into three groups:

1. Premonitory period, characterized by slight difficulty in starting stream, increased frequency, more marked at night, due to a passive congestion of the trigone and prostate and a reflex polyuria. During this stage there is no residual urine.

2. Stage of insufficiency, characterized by partial retention of urine and increased frequency, during day as well as night. This stage will continue on to one of complete retention.

3. Stage of incontinence, bladder becomes distended, sometimes up to the umbilicus, sphincter relaxing every few minutes, allowing a few drops of urine to escape.

Dilatation of the ureters, back pressure on the kidneys, with a resulting cystitis, pyelitis and pyelonephritis come from this over-distended bladder, and the patient suffers from urosepsis, as shown by a slight temperature, a dry, brown tongue, stomach disturbances, a dry, scaly skin, and a sluggish mentality.

The diagnosis is made by rectal palpation of prostate, determination of amount of residual urine and a cystoscopic examination.

Before operation is decided upon the kidney function must be determined. The Phenolsulphonephthalein test is the best. Where the function is low, it must always be brought up by continued preoperative treatment, which consists of permanent bladder drainage by means of a retained catheter; after the bladder has been emptied slowly, daily irrigations of silver nitrate, 1-4000, till cystitis clears up, forced water and urinary antiseptics.

The two-stage operation is reserved for a few cases which become intolerant to the catheter, but it is to be condemned on account of the sloughing wound and the adhesions around the bladder.

Choice of operation: Where finger cannot reach the upper border of prostate or there is marked intravesical enlargement, syprapubic prostatectomy is indicated.

Where prostate is not much enlarged and placed low down, the perineal operation is chosen.

Post-operative treatment consists in the use of the Murphy drip and the electric baker till kidneys functionate. Water should be forced by mouth as soon as patient can swallow.

Tympanites is treated by strychnine and eserin, piturin or hormonal internally, and turpentine stupes over abdomen. Hiccough is controlled by the use of atropin. Stomach lavage is very useful where the vomiting is severe.

Hemorrhage at the time of operation is controlled by a firm tampon in the cavity left after prostate has been removed.

A dental rubber dam dressing helps collect the urine from a suprapubic wound and makes the patient a great deal more comfortable.

Mortality rate varies from 10-40 per cent. With good pre-operative treatment it should be about 10 per cent.

At the Long Island College Hospital, in the past three years, forty-three operations; six deaths. One due to carcinoma of the stomach, four weeks post-operative; one due to cardiac trouble, six weeks post-operative; and four due directly to operation because of poor renal function.

H. V. Raycroft, M. D.

### PROPHYLAXIS AND TREATMENT OF ARTERIOSCLEROSIS.

By Edwin W. James, M. D., Tacoma, Wash.

If we accept, as indeed we must, the statement that no measures will serve to remove calcarous deposits of fibrous tissue from the walls of blood-vessels, once degeneration has taken place, then prophylactic measures assume their proper perspective and demand recognition during a study of this subject. The whole question of preventive medicine is of absorbing interest and, while at first glance it does not seem to have to do largely with arteriosclerosis, who shall say how far-reaching the effects of blotting out preventable diseases may be upon the incidence of arterial changes. For is it not true that typhoid fever, diphtheria, influenza and other infections are prominent causes? And, if inflammatory rheumatism be a common cause, may we not acknowledge our indebtedness to specialized surgery for preventing in a large measure "rheumatism," and so arteriosclerosis, by removal of diseased tonsils? We believe that the coming generation will, in this respect, as in many other more spectacular ways, reap benefit from the well-directed efforts of our public health workers.

Arteriosclerosis is not a disease entity, but a syndrome, a result. It is apoplexy in its possibility, and Bright's disease in its incipiency. It is many times as common now as it was a half-century ago and, in spite of the gradual withdrawal of infectious diseases from among the causes, its frequency will increase unless prophylaxis be given the attention it deserves. The rapid pace we travel, calling as it does for absence rather than presence of physical effort, is telling on this nation, as witness the forced withdrawal from fields of great usefulness of many prematurely broken men. The ancient Romans, as their star came into its ascendency, were ardent athletes, all able-bodied men, especially soldiers, being participants in games and sports. It is told of the most notable warriors of that time that they never missed a day in carrying out their exercises, and were able to successfully contend with any member of their troups. And away back in Roman history we find the decline of athletes coming at the same time as the increase of luxury, athletes being then abandoned to the class of professionals, and finally being taken up by paid combatants or gladiators so familiar in literature. Here in America there seems to be a very large class of men who are consuming the food of athletes and taking their exercise by proxy. The massing of humanity into cities, the short distances necessary to go to transact business and the easy means of transportation for either short or long distances are among the things that have to be considered. Time was when it was considered genteel to walk or ride a horse, while now the effort of holding a steering wheel or changing a tire is something to be guarded against.

Prophylaxis, when to begin. If we attempt to prevent arteriosclerosis at the time when the best results may with confidence be expected, we shall look to the care and education of our youths and maidens. A system of athletics which relegates the exercise and sport to a chosen few will not produce a high degree of physical perfection in the mass of students. Wearing a chrysanthemum to a Thanksgiving day football game never equalized a young man's circulation, although cheering may increase his air-space. However, we have not been aware of any deficiency of air-space in Young America.

All young people should be trained in systematic exercise and sports, for two reasons: First, to develop a strong and healthy body, which will resist invasions of pathogenic organisms, by having the right sort of blood, and blood-vessels; second, they should receive this training in order that they may acquire a taste for out-door sports. It is just as important that a man or woman should know how to play as to know how to work. We have had several cases of beginning arterial change with symptoms, when proper living, with some relaxation, would have prevented further development of the trouble and spared the man to years of continued usefulness, but he did not know how to play. He could not relax, and he had no taste for recreation. That we found to be one of the great difficulties in handling these cases. It is hard to teach a man or woman past middle life to play. So, I repeat, we must see to it that our young people acquire a taste for those things which they will need to do in more advanced years.

Without wishing to introduce any discussion of political policy, but strictly from scientific reasons, we would urge compulsory military training in our public schools and colleges. The physical exercises and the taste of camp life tend toward bodily vigor, mental acumen and a fondness for outdoor life.

It is, however, in connection with middle age that the prevention of arterial changes is most often considered, and at this time of life we find the greatest degree of bodily insult and abuse. Among the well-to-do there is a growing disposition to consult a physician at more or less regular intervals for a physical examination. This is as it should be, and must be encouraged in every way. The doctor who at these examinations fails to go carefully into the habits and mode of life of his patient is derelict in his duty. One should correct errors of living just as surely as errors of digeston or vision, and charge a fee for so doing, for it is a service of even greater value.

We shall do much to prevent arteriosclerosis by, in no uncertain terms, advising these patients of a proper diet and a necessary amount of exercise. Here in the West, where there is so much of God's out-of-doors, people get away for the right kind of outings more than in the East. Not always, however, the people who most need it. We have seen patients presenting distressing symptoms, whose table was too generous and whose exercise was neglected, who have made spectacular recoveries, not under internal medication, but from regular gymnasium work. Golf or other not too vigorous out-door exercise has helped others equally as much. At the same time their intake of food was curtailed. These were cases of Huchard's "preclerosis" or Von Basch's "latent arteriosclerosis." There is a wonderful field for education in the matter of eating. Witness the lunches which we see consumed at any of our clubs or hotels by men who will ride away to their offices and remain physically inactive the remainder of the day.

Alcohol and tobacco should be reduced, if used to excess, or entirely cut off. It is our opinion, however, that there is far greater harm from the intemperate use of food, than from the temperate use of alcohol or tobacco.

Infected tonsils as well as all other foci of infection should be removed, even in the later years of life, as they should in the prophylaxis of many diseases. Acute infections are becoming less common, but should be further guarded against, for they all leave their marks in the arteries, even of young children. Syphilis must be cured before patients are allowed to discontinue treatment. Many cases of atheroma will in this way be prevented. Happily the later antisyphilitic remedies are very great aids in the cure of the disease, and later laboratory methods make known indications for specific treatment or its discontinuance.

The treatment of arteriosclerosis seems to have been enveloped in something of a haze, which no doubt is due to an endeavor to keep away from symptomatic treatment and empirical practice, both of which have a decided place. Furthermore, it is impossible to be at all comprehensive in a study of treatment without some fairly well defined classification of cases. Sir Clifford Allbut has offered the following suggestion which has been accepted by Sir William Osler and others:

(1) Arteriosclerosis, being the effect of persistently high blood-pressure, Allbut's hyperpiesia, Huchard's preclerosis.

(2) Arteriosclerosis of toxic origin from poisons or disease.

(3) Arteriosclerosis of advancing age, the result of involuntary changes, termed by Allbut, decrescent.

It is in the case of hyperpiesia that treatment offers the greatest encouragement, it being possible many times to achieve recovery. Recovery not by removing deposits from the arteries, but in those fairly early cases, by relieving the conditions which later will cause degenerative changes. There may be more or less serious symptoms causing patients to seek advice, or the condition of hyperpiesia may be recognized at the time of insurance examination or when prescribing for some concurrent ailment.

The treatment of hyperpiesia will depend upon the condition of the patient at the time of observation and the exciting cause. The success or failure of treatment will depend not only upon the time at which treatment is begun but upon the co-operation we receive from the patient and his family. There is probably no condition in which a clear conception of psychology is of greater assistance than here. Discipline is an essential and suggestion an aid. Sometimes, perhaps, we err in disregarding the fact that there are two kinds of suggestion, positive and negative. We give the one intentionally and hopefully. Our patient acquires the other, if we are not on our guard, from our actions, facial expression and nature of our advice. The layman nowadays is pretty well posted in blood-pressure, hardening of the arteries, threatened apoplexy, etc. (or let us say he thinks he is), and it is very easy to launch him on a debauch of sordid self-examination and introspection, by awakening apprehensions through too great diligence in examinations and the imposing of too many restrictions.

Have we not sufficient evidence that blood-pressure is raised by worry and nervousness? We have seen a man of fair robustness reduced to semi-invalidism by being told daily for a short time of his blood-pressure which registered about 200. Just as an X-ray plate in the hands of a patient may be a source of great concern, so the knowledge of his blood pressure may be a source of great worry. Both should be kept in the physician's hands. Blood-pressure should not be taken more frequently than the exigencies of a case demand.

A very bad effect will also usually follow the advice to a patient who is still fairly active that he stop all work and responsibility in connection with his business. The reaction will be worse than the continuance in more or less moderation. However, restrictions must be placed and modes of life changed but the very radical changes can be brought about gradually in the mild cases. Having inspired a patient with confidence, instead of apprehension, we are in position to outline treatment for him with fair prospects of success.

Hyperpiesia of children is usually from gastro-intestinal disorders and restriction of diet, followed by the administration of mercury, proves sufficient ordinarily. In treating hyperpiesia of adults the first consideration is that of diet and exercise. We say diet and exercise, believing it impossible to consider either separately. Most men are burning too rich a mixture. Food values must be reduced to terms of muscular exertion. If these people who are so fond of eating would take sufficient exercise to burn up the fuel, the unfortunate results would not follow. And so, while the question of diet is the most important, it must be handled with due consideration to exercise. We believe it is far better to insist on exercise up to the point of safety, with a less restricted diet, than to tolerate inactivity on the low diet.

The exercise which should be prescribed for a man of fitfy, with a systolic blood-pressure of say 160 and diastolic 90, whose heart is slightly enlarged to the left, and who has no symptoms of myocardial insufficiency but is in fairly good condition otherwise, would be those which do not require any sudden or violent exertion. Golf, horse-back riding, walking and graded hill climbing. The English use hill climbing for its therapeutic value much more than we do, and it has been dubbed by them "the terrain cure for rising blood-pressure." Hill climbing should be undertaken gradually at first, for during the first few minutes both systolic and diastolic pressure rise. Then comes one's "second-wind," due to an increase in the lumen of the arteries and consequent relief of the circulation.

Exercise is of value in even more advanced cases after the heart has regained compensation.

The question of diet presents many difficulties. It must be reduced to meet necessary requirements, which usually means deprivation to the patient who, during middle age and inactivity, has been eating about the same as he was at the age of strenuous youth. Reduction to about one-half often gives splendid results. It is generally not necessary to adopt an exclusive diet, rather can we expect better results from a temperate amount of a general diet, at the same time avoiding what we might term the notoriously harmful foods. Meat only in small amounts two or three times a week, no hot cakes or hot breads; no friend food, no pastry, no highly seasoned food and little salt. Much has been written about purin-free diets but clinically we have found no advantage in it. While Brault states that, inasmuch as atheroma is found in the herbivora a vegetable diet is dangerous. Huchard says that meat is a poison to the hyperpietic.

The intake of fluids should be reduced in some cases. Much harm is being done nowadays by the advertisements in the press, urging the universal drinking of large amounts of water, always, of course, adding some patent medicine in the guise of a harmless cure. In this way they say we may "wash out the system" or take an "internal bath." Any excess in the use of alcoholic drinks or tobacco should be foresworn. Life for these patients must be made to run smoothly, avoiding as much as possible mental excitement and worry, while not going to the extreme of forbidding all endeavor and responsibility.

The only electrical treatment which seems to be of value is the high frequency current. One thousand milliamperes should be given for fifteen to twenty minutes. The systolic pressure is often reduced noticeably and remains down for from several hours to two or three days. It is a measure of some usefulness in cases in which high pressure is the cardinal symptom, particularly in nervous people. It acts as a nerve sedative and probably reduces peripheral resistance. Some have claimed that its only good effect is through suggestion. Granting that it is a help in this way, there seems to be plenty of evidence that it does more.

In more advanced cases, with failure of cardia ccompensation as evidenced by dyspnea, edema, and a murmur at the base of the heart, the treatment becomes more active. Such patients must have rest in bed until the heart is able to regain its muscular tone. Digitalis here becomes our drug of choice, administered now as digipuratum. In case the situation is threatening, it is best given intravenously, one dose of one and one-half grains, repeated in twenty-four hours, if necessary. Ordinarily, however, it may be given by mouth, one and one-half grains three times a day for three days, then one-half the dose. We have given this latter dose continuously for months, with only occasional periods of two or three days without it, with no untoward effect, but on the contrary continued improvement.

If there be much edema and rest, diet, cathartics and digitalis do not give relief, we have tried apocynum cannabinum with quite remarkable results in some cases. A fresh tincture is procured and fifteen drops given three times a day, increasing one drop to each dose a day until therapeutic limit is reached. The mechanical removal of fluid may be necessary. Such indeed is indicated if there be anasarca.

Those cases of myocardial insufficiency due to the strain of hypertension with, perhaps, coronary sclerosis, offer a great deal of encouragement from treatment. These patients have kidney involvement but it is secondary. Primary kidney cases do not respond so well to treatment.

Vasodilators are sometimes of value in reducing high pressure. To be sure, it is symptomatic treatment, but why not? A vicious circle may be broken by improvement of a symptom. The nitrites may be used for relief of high pressures, not, however, if the heart shows signs of yielding. If used, it should be in increasing doses and with watchfulness. An occasional course of calomel or blue mass is usually indicated. The use of iodides in other than syphilitic cases is used empirically and with good effect, its favorable action probably being due to a decrease in the viscosity of the blood. This latter is also effected by the use of mineral waters, baths, and purgatives.

Venesection is a remedy very useful in two ways; first, as a part of our treatment in cases of hyperpiesia in robust, plethoric individuals, even in the absence of an emergency; second, as a relief in a crisis. We have used it in both classes to our satisfaction generally. When used to relieve the ordinary symptoms of high pressure in properly selected cases, it is resorted to about twice a year. Following the withdrawal of a pint to a pint and one-half of blood, the patient experiences considerable relief. The blood-pressure drops and may remain at a lower level for several months. It is gratifying to have a patient return in, say, six months for another venesection with the voluntary statement that she has been so much better but feels that it is time to repeat it. Patients who have experienced this relief look forward to the treatment with confidence. In crises like threatening cerebral hemorrhage the results of blood-letting are very encouraging. In choosing cases for venesection we should have clearly before us the classification of arteriosclerosis, for, while it is a measure of considerable value in the hyperpietic form, it is scarcely applicable to the decrescent cases. In fact, the results in the latter class might be disastrous, as also in cases anemic from chronic nephritis.

In toxic arteriosclerosis the blood-pressure is not raised, so depressor remedies are not needed. The treatment is that of the particular poison or infection concerned. Syphilis is the most common infection and a discussion of its treatment could not be undertaken in a paper of this scope. With the present improved remedies at hand and the improved laboratory methods of accuracy in diagnosis, cases of syphilis will generally be cured before sclerosed or atheromatous blood-vessels require treatment. For the toxic forms we may say that rest, massage, baths, diuretics and cathartics will be the remedies used.

We have tried to show that much can be accomplished in the treatment of arteriosclerosis due to hyperpiesia by treating the high blood-pressure to which it owes its origin. Unfortunately, in decrescent arteriosclerosis we can not anticipate any such fortunate results of treatment. It is to this form we refer when we say "a man is as old as his arteries" and, as we can not subtract years from his age, we can not extract calcereous deposits from his arteries. We may watch and wait, lending a hand here and there, but the result is inevitable. We dare not interfere with a moderate rise of blood-pressure, for it is compensatory. The arteries are less resilient and there is more friction, consequently the heart's action increases in force to overcome the odds.

Just a word in regard to indications for treatment, furnished by blood-pressure readings. Diastolic as well as systolic blood-pressure should be taken and the pulse pressure estimated. The probabilities of loss of cardiac compensates the increase in pulse-pressure.

Consequently, a high pulse-pressure is a contraindication for the use of depressant measures. On the other hand, it is a positive indication for guarding the heart muscle and the use of a drug of the digitalis group. In high blood-pressure cases, if the pulse-pressure be low, that is, if the diastolic pressure be comparatively high, cerebral hemorrhage is to be feared and sudden muscular effort might be fatal. Depressants or venesection in such cases may be indicated, although all measures for immediately lowering blood-pressure should be used very guardedly. It is a grave error to assume that every high blood-pressure (systolic) should be promptly reduced. They are frequently compensatory and disastrous results may follow this reduction.—Northwest Medicine.

### CONTROL OF VENEREAL DISEASE.

**By William Warren Townsend, M. D., F. A. C. S.**
**Rutland, Vt.**

This subject is so extensive as to transcend by a wide margin the limits allowed the speaker, so that a number of its more familiar aspects must be left out of consideration as being taken for granted on all sides. Thus, instruction of youth in normal sexual physiology and hygiene is not only useful in itself but exerts a favorable suggestive influence. No one denies that continence is the safest preventive of venereal infection, or that alcohol promotes sexual demoralization. It is only too apparent that insufficient wages for girls makes for unchastity, and it can be shown abundantly that prostitutes are largely recruited from degenerate stock and are very often feeble minded and hence should be under state supervision for such reasons above. The subject of commercialized vice or "white slavery," can be touched on but lightly.

But on many other aspects of the problem there is much illusion and open antagonism. Sanitary prophylaxis is by no means necessarily moral. It is often highly efficient, so much so that it invites and fosters immorality. On the other hand moral prophylaxis may not only be of low efficiency but tends constantly to create new problems which in turn call for solution. But Anglo-Saxon and American public opinion will not condone any sanitation which has not a foundation in morality. Let us make the distinction clear. If a respectable, married woman contracts syphilis innocently from an infected infant (this has often happened to wet nurses) no one would call it immoral to protect her husband from infection by proper enlightenment; but if the infected woman was of loose habits her paramours should be earnestly warned, but on no account instructed to protect themselves in any other manner than abstinence. Disease is regarded as a penalty for sexual transgressions, and while the innocent occasionally suffer this is seen in all offences. The medical man, however, insists that the innocent be spared when there is no moral surrender involved; and there is no serious opposition to treating all infected subjects and at the same time instructing them orally and by tracts as to all measures which bring about a permanent cure, and tell how to avoid contaminating innocent people among whom they are thrown. In accord with the strictest moral canons are all so-called rescue methods which seek to reform loose girls and secure for them legitimate employment. It is not difficult to reach these girls when they are undergoing hospital treatment or under arrest for petty offences. It would be called good sanitation to drive the women out of town but hardly Christian, for they would necessarily pursue their callings elsewhere.

Is it immoral to enlighten the innocent about the venereal peril? Not perhaps when this is done privately. The moralist, however, is sternly opposed to wholesome and indiscriminate enlightenment because of its great suggestive possibilities for evil. Someone has said, instruct in physiology perhaps, but never in pathology. But is it really the moral sense which is outraged by enlightening the innocent? Is it not a mixture of conventionality and prudery? Macaulay held that morality is not fixed but progressive. We make discoveries in morality as we do in science. As soon as it can be shown that enlightenment of the innocent does great good, or even that it does no harm, it will not transcend a moral foundation. As will be seen, publicity in its various aspects will indirectly enlighten the innocent and also protect them.

Before dismissing the conflict between efficiency and morality it is well to speak of the new remedy salvarsan which was heralded as able to cure a person permanently of syphilis in a very short time. The idealist saw in the use of such a remedy a moral surrender. It is true that knowledge of this substance has robbed syphilis of much of its "frightfulness," but a person thus cured readily contracts the disease anew.

No true medical man would ever on any pretext refuse to anyone whatever the benefits of his art.

Our chief study is to lay stress on all practical measures which do not raise the question of sentiment. We have already referred to one—the treatment and enlightenment of all possible patients as near after infection as practicable. This method involves no hardships in personal liberty, such as quarantine and notification. If we take for granted that most victims are young working people of very limited means we must have evening dispensaries but, of course, not for venereal diseases alone. These would not interfere with any other charities, but would supplement their activities. There should be such an institution convenient to a fixed number of people. To give details of a plan which we have in mind for the State of Vermont would require too much space. The service should be in the most competent hands with all necessary equipment, the patient should be treated like any other sick individual, with consideration, and should be supplied with leaflets for enlightenment. These patients should be urged to send their friends and acquaintances. The treatment of private patients will be discussed later.

The real inwardness of the dispensary plan of treatment lies in its attempt to reach the patient when he is in the most infectious state of the disease. By proper treatment and enlightenment he can conserve both his own and society's welfare, for at this period he is a menace to his associates in shops and factories. The compulsory use of the individual drinking cup and similar measures have doubtless done something toward protecting the public. Of leaflets used, one instructs as to gonorrhea, the other as to syphilis. There is nothing in them which can be construed into a license to sin. The plan outlined is an old one and the chief reason against its success has been the expense involved.

The attitude of the private practitioner or family physician has not been what it should have been in the past. Many men high up in medicine either refuse some patients outright or get rid of them by demanding a prohibitive fee in advance. They thus have no occasion to enlighten the patient, who, turned away, may seek a physician of mediocre attainments, a druggist or advertising specialist. The practice of many doctors who accept these cases is to charge a fixed sum and then discharge the patient as soon as the active symptoms have disappeared. In many cases the doctor himself needs enlightenment, since he unwittingly neglects to instruct these patients as to future contingencies. On many occasions these practitioners have sanctioned the marriage of men who were past their active symptoms but very far from cure.

This subject leads us naturally to the problem of the advertising quack, many, if not most, of whom are of the same type as the loan shark. They not only make no effort to cure their patients, but even strive to keep them ill and make them worse whenever by so doing they can extort more money from them. These quacks depend chiefly for their success on newspaper advertising and the best way to expose them is through the initiative of ethical newspapers of wide circulation. It is said that the custom of rejecting quack advertising matter originated with the Sacramento Bee, but the most influential champion of this movement is the Chicago Tribune, which we shall have occasion to mention later. It is said that the Advertising Men's League refuses to handle advertising of this class, not so much on moral grounds as for business reasons, because in the long run it does not pay, one reason being the instability of the quacks, the precarious nature of their activities calling for frequent change of residence. Laws against quack advertising have thus far been placed on the statute books of three states,—Oregon, Massachusetts and Minnesota,—but the movement is not making the headway that it should. A bill to suppress this form of advertising was recently defeated in New York because too drastic. New York City quacks still spend from $100,000 to $200,000 annually in the advertising columns of 16 papers, but one of which, however, is an established daily with large circulation. In certain states the statutes so read that the licenses of advertising quacks are automatically revoked. By far the best plan of killing this industry is the appointment of a Postmaster-General who can or will exclude from the mails any periodical which contains advertising of this class.

Where large bodies of young men are under strict discipline, as in the U. S. Army and Navy, the necessity of efficiency causes venereal diseases to be regarded in the same light as other contagious diseases. While continence is encouraged and fines imposed for sick leave, the men are instructed to protect themselves and given preventives. The results have been excellent, especially in the Navy. It is said that Secretary Daniels objects to the moral surrender involved and would abolish instruction and preventives, but doubtless he would not care to go much further; that is, he would not confiscate preventives or restrict shore leave.

It is impossible to consider the prevention of venereal disease without touching extensively on the subject of prostitution. What about segregation? Modern municipal research has shown that a minority—often a large one—of prostitutes live in a segregated area. The so-called "red light districts" may not shelter over 20 per cent. When such districts have been abolished by prosecuting owners and landlords of property, the inmates do not "scatter through the residence districts," but leave town. Through cooperation of municipal and private benevolent interests girls so dispossessed, have in some towns, been aided to go to their homes or have been aided in obtaining employment. This kind of rescue work is somewhat akin to commitment of wayward girls to large reformatories, in which they learn occupations. The supposed scattering of vice through the respectable districts means simply that the former patrons of the red light districts seek out loose women wherever they are to be found. A practical motive for abolition of segregation is to rid the city of the male hangers-on—the pimps, cheap gamblers, petty thieves, procurers, professional bondsmen, political roughs, etc., etc. The old excuse that the labors of the police are greatly facilitated by a segregation district is no longer valid, because abolition of the district automatically leads to the flight of the undesirable citizens.

What is the mental attitude of the prostitute toward venereal disease? While, in the main, commercialized vice regards transmission of disease as bad for business, the prostitute not only shows in many cases a shocking indifference toward infecting men, but may even in a spirit of revenge refuse treatment, so that she may be able to contaminate men by the score. Here we may refer to the well known case of a New Orleans prostitute who had already infected 300 men and was "out for a record" of 500. When we consider the danger of one such woman in a community we have to think of some drastic but legal course to pursue. Laws against wilfully conveying a disease have seldom led to convictions. Certainly since the Vermont law covering this offence has been in force we are not aware that a conviction has even been sought, although in a few instances women have been committed to the Vermont House of Correction charged with disorderly conduct, etc., in order that they might be given treatment for syphilis or gonorrhea. The State law gives the Health Board absolute authority over such women but there is a joker in the form of an appropriation of $1,000 to carry on the work which the representatives of the tax payers evidently regard as too much money.

What have Health Departments accomplished in the suppression of venereal diseases? We can only consider a few of the plans and results. Cincinnati began about 1900 with a system of weekly inspections and health certificates with fees. This was done with the consent of the examined. Healthy women were certified, while diseased ones were interned and certified upon recovery. This plan lessened venereal infection to the extent of 5 per cent. or 10 per cent,—low efficiency plus moral surrender. Weekly inspections have only a limited value for various reasons. Many women are so-called "carriers,"

i. e., they can transmit a disease while apparently healthy.

About 1906 the now well-known Cincinnati plan was introduced. Instead of inspectors and certificates of health the contagious disease placard was placed on any brothel which sheltered an infected inmate. As a result the proprietors at once sent all diseased girls to the hospital. The plan also included the systematic reporting of all cases of venereal disease as being contagious affections. In New York City reporting has shown the fearful prevalence of gonorrhea and syphilis, for of course only a minority of these diseases are placed on record. In Vermont the wording of the statute which provides for reporting by ''physicians who know,'' has driven many patients to consult quacks outside the state or local druggists. This error could be banished by making it compulsory for ''any person who knows,'' etc. Despite this drawback there have been reported in 41 months in Vermont, 901 cases of gonorrhea and 604 cases of syphilis (personal communication from the Secretary of the State Board of Health). In Cincinnati reporting is based on inspection visits made jointly by district physicians and sanitary inspectors to prevent collusion and the sale of fraudulent certificates.

The somewhat drastic plan of abolishing segregated districts by legal prosecutions of owners and lessees of property, known as the ''Chicago plan,'' is said to have worked well, but is comparatively recent and doubtless the forces back of prosecutions will vary with the result of municipal elections. In any case opportunity is afforded to come in contact with the prostitutes who become public charges and can be interned and treated when infected and when sound can be either sent home or given employment or placed in charge of rescue missions or otherwise dealt with for their own and the communities' good.

An extensive problem connected with prostitution is street solicitation and in general the activities of the ''free lance'' as distinguished from the ''white slave.'' The subject of white slavery we must omit for lack of space, and because there are many old laws which are able to deal with it. Commercialized vice flourishes in small hamlets as well as in great cities, but the free lance far outnumbers the brothel inmate and is responsible to no one. White slavery degrades a woman to the lowest depths but the slave is less a public eyesore and a source of infection than her free sister, who in addition to open solicitation comes and goes as she pleases in saloon back rooms, cheap hotels, dance-halls, steamboats and all public resorts. An old rural problem which still exists to some extent in Vermont is that of the "Swamp Angel," the prostitute who plies her trade in the woods.

Small cities vary greatly in regard to prostitution. One will pride itself in having no brothels while several may flourish in a neighbor town. But in all of these towns there will be found plenty of loose women.

The line is drawn very thinly between the prostitute and the merely immoral woman, for the latter usually profits by her immorality.

There is very little literature on suppression of venereal diseases in small communities. Quite recently Williams and Brown have described local conditions in Montclair, N. J. (Medical Record, December 6, 1913). The average man is panic-stricken about syphilis. He overrates its malignancy just as he underrates the malignancy of gonorrhea. "Fear of syphilis" is a strong incentive to virtue. Early marriage should greatly reduce the number of venereal infections, but as matters stand to-day there is an increasingly long interval between sexual development and marriage, which makes for illicit relations. In communities like Montclair the clergy and church laymen are partly in accordance with and partly opposed to the medical men. The strong point of the former is abstinence up to the time of marriage, and in any case compulsory certificates of freedom from disease. But a new problem is thereby raised, that of the man condemned to celibacy by such a course. It is no simple thing to determine whether or not a man is permanently free from venereal disease and the conscientious physician would often feel like postponing the granting of a certificate. The expense of examinations will usually prove much greater than the fees allowed. In small neighborhoods refusal of a health certificate might easily leak out and ostracism would force the men to seek other regions. Grounds for heavy damage suits might sometimes be present. It is sometimes held that revelation of disease in men about to wed is a blessing in disguise, because the victim will submit to treatment and will henceforth live a rational and hygienic existence and that the postponement of marriage need not be a hardship. No doubt the custom would work both ways. Time alone can decide this problem, as applied to small communities.

One of the most important problems in connection with the control of venereal disease is the prevention of blindness. This is due to direct contamination of the eyes of the newly born child by the infectious secretions of the maternal birth-passages, and is generally held to be prevented by systematic applications to the eyes of the child by the obstetrician, nurse or midwife. The problem is by no means as simple as it appears, if the remedy is applied indiscriminately to all new born babies. Perhaps it would be better to individualize the applications as to strength, time and state of the eyes at birth. There is no doubt, however, that the routine application of the remedy in question greatly lessens blindness in certain communities. With us in Vermont, the State furnishes a neat package containing a solution of nitrate of silver for routine use by obstetricians.

The chancroid or third venereal disease, by reason of its local character and infrequency, presents no social problem whatever and is not included under the venereal peril. As a rule it occurs in the lowest and filthiest of women, but notable exceptions occur. During the present war it has been discovered that the micro-organisms which causes chanchroid may sometimes be found in the secretions of a woman not herself infected. A slight abrasion during sexual congress produces a port of entry for this filth organism and the man develops a chancroid just as if the woman herself were infected. Among soldiers chancroid can cause considerable loss of efficiency. In Vermont chancroid is very rare and is usually imported. With the return of a cavalry regiment from the tropics several years ago we saw many chancroids and heroic treatment was necessary to arrest the local destruction of tissue.

In concluding, reference should be made to the subject of newspaper publicity on matters connected with control of venereal disease and its application to its smaller communities. The Chicago Tribune after it had refused quack advertising made the daring experiment of publishing on its first page, matter concerning the venereal peril. One object was to learn whether its circulation would suffer. Several years have now elapsed and the paper announces that its circulation has not been impaired. Such a course would be too venturesome for the dailies of a small city, but metropolitan papers have an increasing tendency toward out-of-town circulation and many small communities may in turn benefit by this plan. Even here, however, there could be a clash with men of the Comstock type who might attempt to exclude such matter from the mails as being indirectly demoralizing, by causing the innocent to think about vicious subjects.—The Urologic and Cutaneous Review.

